# Comparative efficacy of Chinese herbal injections for septic shock: A protocol for systematic review and network meta-analysis

**DOI:** 10.1097/MD.0000000000028183

**Published:** 2022-02-04

**Authors:** Peiying Huang, Yan Chen, Qiang Liu, Sisi Lei, Yuchao Feng, Qihua Wu, Haobo Zhang, Bojun Chen, Zhongyi Zeng

**Affiliations:** a The Second Clinical Medical College of Guangzhou University of Chinese Medicine, Guangzhou, China; b Department of Emergency, The Second Affiliated Hospital of Guangzhou University of Chinese Medicine, Guangzhou, China; c Emergency Department of Guangdong Provincial Hospital of Traditional Chinese Medicine, Guangzhou, China; d Shenzhen Traditional Chinese Medicine Hospital, Shen Zhen, China.

**Keywords:** Chinese herbal injections, efficacy, network meta-analysis, septic shock

## Abstract

**Background:**

Septic shock is a life-threatening syndrome. Despite Western medicine guidelines being continually updated on septic shock, the disease still has a high mortality rate. Chinese herbal injections (CHIs) are injections made from effective components of traditional Chinese medicine, which have a potential therapeutic effect on septic shock and are recommended as the adjunctive treatment for septic shock in China. Although pairwise meta-analysis has been published for category-single CHIs about treatment effects of septic shock, there is no meta-analysis comparing more than 3 various types of CHIs used for septic shock.

**Methods:**

Chinese and English databases will be retrieved for randomized controlled trials from the establishment of the databases to September 30, 2021. Two reviewers will perform literature searches and data extractions while another 2 reviewers for risk assessments. RevMan V.5.4 software, Stata V.14.0 software, and R V. 4.1.1 software will be applied to perform pairwise meta-analysis and network meta-analysis. We will apply the Cochrane risk of bias tool to assess the risk of bias while the Grades of Recommendation, Assessment, Development, and Evaluation approach will be used to summarize the results of the study. The PRISMA-P guideline was followed for this protocol.

**Results:**

The current study will explore the therapeutic effect of CHIs in the treatment of septic shock through pairwise meta-analysis and network meta-analysis.

**Conclusion:**

This study will seek out the best-performed CHIs under various indicators for septic shock, providing supporting evidence for clinical selection of CHIs for septic shock.

## 1 Introduction

Sepsis, an acute syndrome triggered by infection, was further defined as a concurrent state including infection and life-threatening organ dysfunction caused by dysregulated host response.^[1]^ Septic shock was classified as the most severe subset in sepsis, being described severe circulatory failure along with abnormal cell and metabolic function.^[1]^ An epidemiological survey showed that the incidence and mortality of sepsis are substantially worrisome, with approximately 48.9 million cases globally in 2017 in which 11.0 million deaths were reported, accounting for 19.7% of all global fatalities.^[2]^ Septic shock, a comprehensive pathological critical state that has higher mortality of 40% to 60% relatively, is a leading cause of death in sepsis patients.^[3]^

Indeed, since 1991 when Sepsis 1.0 was released, we have experienced an ongoing process of renewal about definitions and treatment modalities of sepsis and septic shock.^[1]^ Treatment options of Western medicine (WM) for septic shock are continually being refined (e.g., from 3-hour, 6-hour, and 24-hour management bundle to 1-hour management bundle).^[4–7]^ However, with improving therapeutic approaches, the definitive decrease of mortality in septic shock remains uncertain.^[8]^ The economic burden of septic shock is growing annually for the presents.^[9]^ It is essential for searching for better therapies or complementary medicines to reduce the severity and case-fatality rate of septic shock patients.

The same as steroids, vitamin C, and thiamine, Chinese herbal injections (CHIs) are also considered as an adjunct therapy for septic shock patients.^[10]^ CHIs consist of active ingredients of single Chinese medicine or Chinese medicine compound, having been confirmed to exert effects in septic shock patients.^[11–13]^ A pairwise meta-analysis reported that Shenfu injection plus WM could decrease 28-day mortality, increase mean arterial pressure and normalize heart rate when compared to standard therapy.^[14]^ In analogy to the study, a combination of Shenmai injection plus WM was significantly more effective at reducing mortality of septic shock patients relative to WM only.^[15]^ Xuebijing injection and Shengmai injection were also be proven to be effective therapies for septic shock patients via pairwise meta-analysis.^[16,17]^Based on this, CHIs have been integrated into the program of Chinese guideline for the treatment of septic shock.^[18]^

CHIs have played a role in the treatment of septic shock, however, in some specific clinical circumstances, how to choose the optimal CHIs is still an unresolved problem. Study has yet compared all CHIs for the treatment of septic shock at present. Consequently, we initiate this study to compare the efficacy of different kinds of CHIs used to combat septic shock in different outcomes, looking for the best performer in improvement of various indicators.

## 2 Materials and methods

### 2.1 Study registration

The study procedure of this protocol was followed the Preferred Reporting Items for Systematic Review and Meta-analysis Protocols (PRISMA-P).^[19]^ We have registered on the International prospective register of systematic reviews with registration number CRD42021282958 (URL: https://www.crd.york.ac.uk/prospero/display_record.php?ID=CRD42021282958).

### 2.2 Inclusion criteria

#### 2.2.1 Types of studies

Randomized controlled trials with one-or-more arms will be included, whereas cross-over trials were not eligible given the impacts of interventions in prodromal phase. We will not set restrictions on language, country, date of publication, and stage of original researches.

#### 2.2.2 Types of participants

Patients of both sexes aged 18 years or older, with definitive diagnoses of septic shock, will be included. We will not limit patients’ diagnostic criteria, for that, there are frequent iterations and updating of WM guidelines in which each of the criteria showed high similarity. Furthermore, no restriction will be put on race and sample size of the patients. Nevertheless, studies targeting patients with concurrent septic shock and severe profiles of comorbidities most likely impacting prognosis (e.g., cardiac arrest and advanced cancer) will be excluded.

#### 2.2.3 Types of interventions

##### 2.2.3.1 Experimental interventions

The experimental group received one type of CHIs in addition to WM, with intravenous administration. We will not make restrictions on time to treatment initiation, course of treatment, frequency of administration, and dosage form of CHIs while treatment protocol complicated with other traditional Chinese medicine (pharmacological or non-pharmacological intervention) will not be considered.

##### 2.2.3.2 Comparator interventions

The control group was treated with WM, such as 1-hour, 3-hour, 6-hour, or 24-hour management bundle with anti-infective therapy. On this basis, adding another type of CHIs will also be considered.

#### 2.2.4 Types of outcome measures

##### 2.2.4.1 Primary outcome

The primary outcome is 28-day mortality. Although the closest estimate to 28-day mortality was included in the study of Daniel et al,^[20]^ it will not be considered in our study.

##### 2.2.4.2 Secondary outcome

Secondary outcomes include:

1. intensive care unit length of stay;
2. hospital length of stay;
3. post-treatment Sequential Organ Failure Assessment (SOFA)score;
4. post-treatment Acute Physiology and Chronic Health Evaluation II (APACHE-II) score;
5. post-treatment procalcitonin level;
6. post-treatment serum lactate level (differences were moderate and non-significant between arterial lactate level and venous lactate level, especially when serum lactate level was less than 4.0 mmol/L.^[21]^ Therefore, we will not make a distinction between arterial lactate and venous lactate).

##### 2.2.4.3 Adverse drug events

The adverse events of CHIs will be presented as descriptive statistics in a form.

### 2.3 Database and search strategy

We will search the following databases: PubMed, Embase, Web of Science, Cochrane Library, China National Knowledge Infrastructure, Wanfang Database, Weipu Journal Database, and Chinese Biomedical Literature Database. The search time will be set from database establishment to September 30, 2021. Ongoing or unpublished studies which have been registered will be considered. The search strategies were developed by 2 of our team members and further decided after the discussion of all team members. The detailed searching term and strategy of PubMed is shown in Table 1. Additionally, to avoid missing relevant studies, the references of the included studies will also be searched via Google Scholar. All the search results will be imported into EndNote V.X9, Clarivate Analytics, USA.

**Table 1:** Detailed search strategy for PubMed.

No.	Search items
#1	Shock, Septic [MeSH Terms]
#2	Shock, Septic[Title/Abstract] OR Septic Shock[Title/Abstract] OR Shock, Toxic[Title/Abstract] OR Toxic Shock Syndrome[Title/Abstract] OR Shock Syndrome, Toxic[Title/Abstract] OR Toxic Shock Syndromes[Title/Abstract] OR Toxic Shock[Title/Abstract] OR Shock, Endotoxic[Title/Abstract] OR Endotoxin Shock[Title/Abstract] OR Endotoxin Shocks[Title/Abstract] OR Shock, Endotoxin[Title/Abstract] OR Shocks, Endotoxin[Title/Abstract] OR Sepsis Shock[Title/Abstract] OR Infection Shock[Title/Abstract] OR Infectious Shock[Title/Abstract] OR Infective Shock[Title/Abstract]
#3	#1 OR #2
#4	Chinese Herbal Injection[Title/Abstract] OR Chinese Herbal Injections[Title/Abstract] OR xingnaojing[Title/Abstract] OR danshen[Title/Abstract] OR shenqifuzheng[Title/Abstract] OR shuxuetong[Title/Abstract] OR huangqi[Title/Abstract] OR tanreqing[Title/Abstract] OR reduning[Title/Abstract] OR xiyanping[Title/Abstract] OR qingkailing[Title/Abstract] OR yiqifuzheng[Title/Abstract] OR yiqifumai[Title/Abstract] OR xuebijing[Title/Abstract] OR shenfu[Title/Abstract] OR shenmai[Title/Abstract] OR shengmai[Title/Abstract]
#5	Controlled Clinical Trial [Publication Type] OR Randomized Controlled Trial[Publication Type] OR Equivalence Trial[Publication Type] OR Pragmatic Clinical Trial[Publication Type] OR random∗[All Fields]
#6	#3 AND #4 AND #5

### 2.4 Study selection and data extraction

**Figure 1:**
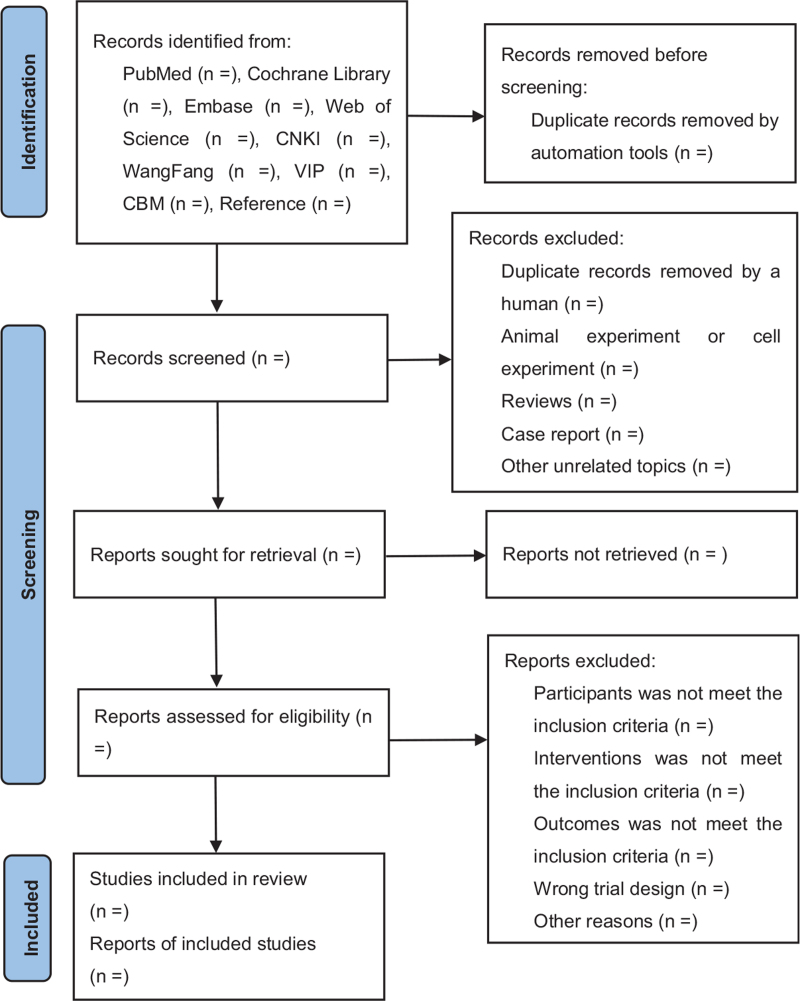
Flowchart of literature-screening process. CNKI, China National Knowledge Infrastructure; VIP, Weipu Journal Database; CBM, Chinese Biomedical Literature Database.

After removing duplicate studies in the retrieved articles, 2 reviewers will screen the records independently by title and abstract reading and then by full-text reading according to the “PICOS” confine (Patient population, Intervention, Control, Outcome, and Study design) we have set in advance. The process of literature screening is shown in Figure 1.

A predefined structured template will be used by the reviewers to extract the following information of the included studies independently:

1. General information (title, year of publication, first-author, demographic, diagnostic criteria).
2. Interventions (experimental intervention and comparator intervention).
3. Outcomes (primary outcome and secondary outcomes).
4. Methodologies (randomized methods, allocation concealment, blinding methods).

Consensus will be reached by discussion between the reviewers if disagreements arise. A third senior reviewer will provide arbitration when the disagreements still cannot be resolved. All data will be recorded in Excel V.365, Microsoft, USA.

### 2.5 Risk of bias assessment

The risk of bias of the enrolled studies will be assessed using the Cochrane Collaboration risk of bias tool by 2 independent reviewers.^[22]^ We will present an assessment of the risk of bias in the included studies as follows: random sequence generation, allocation concealment, blinding of participants and personnel, blinding of outcome assessment, incomplete outcome data, selective reporting, and other biases (e.g., pharmaceutical company sponsorship). Each entry will be rated as “low, “high,” or “unclear risk.” The risk of bias of an enrolled study will be classified as “low risk” only when all the child items were scored as “low risk.” Otherwise, the study will be identified as “high risk” as long as one domain is assigned as “highrisk” or “unclear bias.” The outcomes of the assessment will be presented in 2 forms via RevMan V.5.4 software: a risk of bias summary and a risk of bias graph. Similarly, in the event of disagreement, a consensus will be reached following a discussion by the reviewers. A third reviewer will be available to arbitrate the controversies that remain unresolved.

If missing information is present during the evaluation process, for example, no specific randomized fashion was reported in a study, the author of the study will be contacted for further information. Alternatively, we will rate the term as “unclear risk” if the author of the study cannot be contacted. It is worth noting that the author tends to provide additional information that is favorable to the outcomes of the study. Consequently, the results of the assessment will be discerned with caution.

### 2.6 Statistical analysis

#### 2.6.1 Measures of treatment effect

For categorical variables, we will extract both the number of event responses and the total number of events. Pooled dichotomous-effect measures will be expressed as pooled risk ratio with 95% confidence interval. Meanwhile, we will extract means and standard deviations of continuous variables and merge them as mean differences with 95% confidence interval For studies that expressed continuous variables as medians and interquartile ranges, we will transform them into means and standard deviations.^[23,24]^

We will only compare the post-treatment effects of SOFA score, APACHE-II score, procalcitonin level, and serum lactate level when there was no statistical difference at baseline between the groups, considering the deficient report of the differences between pre-and post-treatment. Although the differences between pre- and post-treatment could be calculated through both pre-treatment and post-treatment effects, it will not be performed to account for the calculation errors.^[22]^ Furthermore, what requires elucidation is that post-treatment effects of day 1, 3, 5, and 7 of partial secondary outcomes (SOFA score, APACHE-II score, procalcitonin level, and serum lactate level) will be collected in our study, and cluster analysis will be performed to pool the effects of different periods in each outcome by a network package in Stata V.14.0, STATA, USA.

Units of the outcomes will be unified, which will be either converted to consistency or excluded, depending on the unit for which the largest number of the included studies.

#### 2.6.2 Pairwise meta-analysis

If there are one or more closed loops between different interventions, pairwise meta-analysis will be performed before network meta-analysis to facilitate the subsequent evaluation of the differences between pooled effects and direct effects.

We will conduct a PMA of each direct pairwise comparison using a meta package of R V. 4.1.1 software, provided that the number of included studies involving 2 interventions is greater than or equal to 2. A more inclusive model, namely, random-effect model, will be used to combine the effects, given clinical heterogeneity existing in the included studies. The results will be shown in a forest plot. *I*^2^, *tau*^2^, and *P* values of heterogeneity tests will be used to further assess the statistical heterogeneity of the included studies, even though we have used the random-effect model. For *tau*^2^, the larger value means the greater heterogeneity among the included studies. If *I*^2^ < 50% and *P* > .1, the heterogeneity is acceptable.^[22]^ Otherwise, subgroup analysis or sensitivity analysis will be conducted to explore the source of the heterogeneity. However, if *I*^2^ > 75% and the source of the heterogeneity is not identified, pairwise meta-analysis should be abandoned and descriptive results will be presented.^[25]^

#### 2.6.3 Network meta-analysis

We will use Bayesian network meta-analysis to merge and further rank the therapeutic effects of various types of CHIs in the current study. A gemtc package of R software will be used to invoke JAGS software and further implement network meta-analysis via a random-effect model. In terms of the random effect, Bayesian meta-analysis adopts a Markov Chain Monte Carlo methodology to construct the model, which is more advantageous than frequentist network meta-analysis.^[26]^

Based on 4 Monte Carlo Markov Chains, we will set the number of iterations as 200,000 and the first 10,000 were used for the annealing algorithm to eliminate the influence of the initial value. Then, trace plots, density plots, Brooks-Gelman-Rubin plots, and Potential Scale Reduction Factors will be used combinedly to evaluate the convergence of the model.^[27]^ If the convergence is suboptimal, the number of the iterations will be increased.

In the presence of more than 2 interventions, we will plot a node network diagram to visualize the relationships among the interventions. If one or more closed loops are formed, tests for assessment of the inconsistency will be carried out:

1. The inconsistency will be preliminarily observed by deviance information criteria between a consistent model and an inconsistent model (different values within five means that the inconsistency is acceptable).^[28]^
2. We will further explore the inconsistency by a node splitting model (*P* < .05 means significant inconsistency).^[29]^

To select the optimal CHIs for each outcome, we will rank each of the pooled effects via a surface under the cumulative ranking area curve and present a league table to show comparisons among each CHIs. In addition, a Global *I*^2^ test will be performed to detect the overall heterogeneity. Moreover, direct, indirect, and mixed heterogeneity between each pair of the comparisons will be sought out by a per-comparison *I*^2^ test. The processing method of *I*^2^ between network meta-analysis and PMA is identical.

### 2.7 Evaluation of publication bias

Funnel plot and Egger test will be used to explore publication bias for the outcomes with more than 10 included studies. If the results are positive, a trim and fill method will be performed to further test the difference in effects before and after correction. Small changes will be accepted. Nevertheless, if there is still strong evidence of publication bias (*P* < .05), we will search for the sources of publication bias or report the pooled results and describe them objectively in the discussion.

### 2.8 Subgroup analysis and sensitivity analysis

The presence of statistical heterogeneity or publication bias is not a prerequisite for sensitivity analysis. We will also detect the robustness of the model by excluding unclear-risk studies, low-risk studies, or studies with obviously clinical heterogeneity respectively. Analogously, in addition to detecting the sources of the heterogeneity or publication bias, subgroup analysis will also be used to assess the robustness of the pooled effects according to:

1. Different diagnostic criteria.
2. Different treatment regimens of WM.
3. Language of the included studies.
4. Time of publication.
5. Type of comorbidities in septic shock patients.

If we find any other covariates affecting the outcomes in subsequent process of our study, we will add extra subgroup or sensitivity analysis based on the covariates.

### 2.9 Grading the quality of evidence

To better promote the results of our study to clinical practice, we will employ the Grades of Recommendation, Assessment, Development, and Evaluation approach to summarize the quality of evidence through GRADE pro software. Randomized controlled trials started with the highest grade, will be downgraded by risk assessment (study limitation, indirectness, inconsistency, imprecision, publication bias), and be rated for the quality of evidence for each outcome: “high,” “moderate,” “low,” or “very low.”^[30]^ Evaluations of evidence will be analyzed from 2 aspects: pairwise comparison and ranking of treatments.^[31]^ All members of the review board will participate in the judgment of the quality of evidence.

## 3 Discussions

The promotion of traditional Chinese medicine compound prescriptions has been restricted worldwide due to unclear compositions and vague mechanisms.^[32]^ However, CHIs have explicit drug components of which part of the mechanism of actions for anti-septic-shock therapy has been elucidated by basal experiments.^[33]^ This study will explore the therapeutic effects of CHIs in the treatment of septic shock patients from the aspects of anti-infection, anti-shock, and prognosis, seeking out the best CHIs in each aspect. We hope that it may be possible to help improve the therapeutic efficiency of septic shock patients.

## Author contributions

**Conceptualization:** Peiying Huang, Bojun Chen, Zhongyi Zeng.

**Data curation:** Peiying Huang, Yan Chen, Qiang Liu, Sisi Lei.

**Formal analysis:** Peiying Huang, Yan Chen.

**Funding acquisition:** Bojun Chen, Zhongyi Zeng, Yuchao Feng, Sisi Lei.

**Methodology:** Peiying Huang, Yan Chen, Qihua Wu, Haobo Zhang.

**Project administration:** Peiying Huang, Bojun Chen, Zhongyi Zeng, Qiang Liu.

**Writing – original draft:** Peiying Huang, Yan Chen, Zhongyi Zeng.

**Writing – review & editing:** Bojun Chen, Zhongyi Zeng.
